# Response to “Comments on Economic Evaluation of Ultrasound-guided Central Venous Catheter Confirmation vs Chest Radiography in Critically Ill Patients: A Labor Cost Model”

**DOI:** 10.5811/westjem.2023.1.60160

**Published:** 2023-03-06

**Authors:** Enyo A. Ablordeppey, Adam M. Koenig, Abigail R. Barker, Emily E. Hernandez, Suzanne M. Simkovich, James G. Krings, Derek S. Brown, Richard T. Griffey

**Affiliations:** * Washington University School of Medicine, Department of Anesthesiology, St. Louis, Missouri; † Washington University School of Medicine, Department of Emergency Medicine, St. Louis, Missouri; ‡ Washington University School of Medicine, St. Louis, Missouri; § Washington University, Center for Health Economics and Policy at the Institute for Public Health, St. Louis, Missouri; ¶ Washington University School of Medicine, Division of Pulmonary Critical Care Medicine, Department of Medicine, St. Louis, Missouri; || Medstar Health Research Institute, Division of Healthcare Delivery Research, Hyattsville, Maryland; # Georgetown University School of Medicine, Department of Medicine, Washington, DC; ** Washington University in St. Louis, Brown School, St. Louis, Missouri

We would like to thank Austin et al^1^ for their interest in our article comparing labor costs of two approaches to central venous catheter (CVC) confirmation,^2^ recently published in the *Western Journal of Emergency Medicine*. In their letter to the editor, they acknowledge that the point-of-care ultrasound (POCUS)-guided confirmation method offers direct and potential indirect cost benefits when compared with the chest radiograph (CXR) method. However, they raise several important points regarding our results. First, we reported a modest difference between direct labor cost of using POCUS-guided confirmation for central lines vs traditional CXR confirmation in our calculations. The authors expressed concern that these conservative cost savings may have less impact in smaller hospitals where only a few hundred central lines are performed annually. The authors state that the cost of added ultrasound machines, formal education of staff, and medicolegal concerns may be barriers to clinical adoption.^3^,^4^ We did find that those were some reported barriers; however; of note, in our manuscript decision tree, there is an assumption that the ultrasound is already available for use since it is typically used to guide *insertion* of CVCs.^4^ The cost of additional training of CVC confirmation has not been measured in any studies to our knowledge, and we agree that perceived medicolegal risk may be a barrier for some institutions or individuals. Our labor cost calculations were the result of conservative salary estimates of a 60-hour work week of physicians using data from 2019 estimates.^5^ We note that the 2021 estimated hourly salaries reported by the authors are higher and can influence calculations.

Second, we only calculated direct cost attributed to physician confirmation and not advanced practice practitioners (APP) as several places may be accustomed. We agree that the direct cost savings of POCUS-confirmation for central lines could be greater when performed by APPs ($10.56 or $10.45), as compared to the CXR method ($18.69). This $4.31 difference between a cost savings of $3.82 (as we originally reported) vs $8.13 (that the authors report) is notable but may not be sufficient to persuade individual or institutional behavior and policy changes. Future studies understanding how facilitators like cost savings drive implementation of POCUS-guided CVC confirmation would be useful.

Finally, although we acknowledge that there is a notable time savings with POCUS confirmation^6^–^8^ contributing to indirect costs, we did not measure them in this study. We are pleased to hear that the critical care resuscitation unit at the University of Maryland Medical Center uses this innovative practice and can pragmatically appreciate the direct and indirect benefits of POCUS-guided CVC confirmation. The fact that a clinician can place a CVC, confirm placement, and initiate care all in one sitting without leaving the patient bedside is an important advantage to POCUS-guided confirmation. Future studies should characterize the resource implications of substituting POCUS-guided CVC confirmation more fully by conducted a comprehensive assessment of the costs of protocol development, implementation, and maintenance of this change in practice.
